# Nephrological factors may cause kidney dysfunction in patients with common variable immunodeficiency

**DOI:** 10.3906/sag-2012-166

**Published:** 2021-08-30

**Authors:** Gökhan AYTEKİN, İsmail BALOĞLU, Fatih ÇÖLKESEN, Eray YILDIZ, Şevket ARSLAN, Kültigin TÜRKMEN

**Affiliations:** 1 Department of Immunology and Allergy, Konya City Hospital, Konya Turkey; 2 Department of Nephrology, Niğde Ömer Halisdemir University Education and Research Hospital, Niğde Turkey; 3 Department of Immunology and Allergy, Meram Faculty of Medicine, Necmettin Erbakan University, Konya Turkey; 4 Department of Nephrology, Meram Faculty of Medicine, Necmettin Erbakan University, Konya Turkey

**Keywords:** Chronic inflammation, proteinuria, common variable immunodeficiency

## Abstract

**Background/aim:**

Common variable immunodeficiency (CVID) is a heterogeneous primary deficiency characterized by hypogammaglobulinemia, recurrent infections, an increased risk of autoimmune disease, malignancy, and chronic inflammation. Proteinuria is one of the most important prognostic factors causing progression in kidney disease. Proteinuria causes tubulotoxicity, activates inflammatory markers that cause fibrosis, and consequently nephropathy progression. The data is scant in the literature regarding the inflammation and nephropathy in CVID. Hence, in the present study, we aimed to investigate the relationship between tubular dysfunction, proteinuria, and inflammation in patients with CVID.

**Materials and methods:**

This was a cross-sectional study involving 27 patients with CVID (15 females, 12 males; mean age, 39.88 ± 13.47 years) and 18 control subjects (10 females, 8 males; mean age, 33.83 ± 7.97 years). Patients were evaluated for kidney functions including glomerular filtration rate, fractional excretion of sodium, metabolic acidosis, serum/urine anion gap, 24-h urine proteinuria and, were grouped in terms of proteinuria. Blood samples obtained from the patients with CVID were taken into 2 mL EDTA tube to evaluate peripheral NK cell subgroups according to CD56 and CD16 expression and CD3, CD4, CD 8 expression to determine subtypes T cells. These cells were evaluated by flow cytometry technique.

**Results:**

Urinary density, fractional excretion of sodium, proteinuria, and metabolic acidosis are found to be higher in patients with CVID when compared to healthy controls. In the bivariate correlation analysis, proteinuria was positively correlated with age (r = 0.496, p = < 0.001), CD8+T cells percentage (r = 0.427, p = 0.02). Albumin, CRP, and CD8+T cell percentage were found to be independent variables of proteinuria.

**Conclusion:**

Increased chronic ongoing inflammation was found to be associated with proteinuria in patients with CVID. Hence, in routine outpatient clinics, proteinuria should not be overlooked in this group of patients.

## 1. Introduction

Common variable immunune deficiency (CVID) refers to a group of heterogeneous disorders that are often due to inherited defects of the immune system. Patients with CVID are frequently susceptible to recurrent infections, autoimmunity, lymphoproliferation, and malignancy [1]. Because of the perception of CVID as rare congenital diseases, heterogeneity of diseases, and lack of awareness, delay in diagnosis is frequent. Partial elimination of problems in access to immune replacement therapy, effective treatment of infections with antibiotics, and introduction of targeted monoclonal antibodies have significantly reduced mortality in CVID patients. As a result, the management of the complications and the quality of life has become more important with increased life expectancy in CVID patients. Although recurrent and widespread upper and lower respiratory tract infections and increased autoimmunity, lymphoproliferation, and especially increased lymphomalignies are well-defined complications of immunodeficiencies, renal complications are relatively rare and overlooked in this group of patients [2].

Increased inflammatory markers such as C-reactive protein, interleukin (IL)-1, IL-6, and tumor necrosis factor (TNF)-α, interstitial cellular adhesion molecule-1, vascular cellular adhesion molecule-1, CD8+T cells, natural killer (NK) cells, and E-selectin are associated with the development of nephropathy in many chronic diseases [3]. Inflammation and inflammatory molecules are also thought to affect glomerular functions through alternations in vascular permeability, vasodilator, and vasoconstrictor mechanisms, extracellular matrix dynamics, and the proliferation of mesangial, endothelial, and vascular smooth muscle cells, as well as the induction of cytotoxicity, apoptosis, and necrosis in the pathogenesis and progression of chronic kidney disease (CKD) [4].

Proteinuria is one of the most important prognostic factors causing progression in kidney disease. Proteinuria causes tubulotoxicity, activates inflammatory markers that cause fibrosis, and consequently nephropathy progression. Most studies have found that better renal outcomes are associated with agents that lower proteinuria [5,6]. For this reason, depending on the underlying etiology, reduction of proteinuria, by the renin-angiotensin-aldosterone blockade, immunosuppressive treatment, and diet regimens, is one of the important parts of the treatment to slow the progression [7].

To date the data regarding the nephropathy in CVID is scant. Hence, we aimed to investigate the relation between the tubular dysfunction, proteinuria, and inflammatory cells including T and NK cells in patients with CVID.

## 2. Material and methods

The study group included 27 patients with CVID (male (M): 12 (44.4%)/female (F): 15 (55.6%), age: 39.88 ± 13.47) who follow up at regular basis and 18 patients (F/M: 10/8, age: 33.83 ± 7.97) as a control group. The study protocol was approved by the ethics committee of the university (sate: 16.11.2018; approval number: 2018/1574). Informed consent was obtained from study participants. The diagnosis of CVID was made according to the updated diagnostic criteria of ESID [2]. 

Demographic and clinical data were retrieved from individual medical files, which all recorded at the first visit of patients including, sex, age, diagnostic delay, detailed family history, and all other necessary information. Initial immunological workup and other diagnostic investigations to expose exact diagnosis and concurrent complications and/or disorders were also recorded. 

Venous blood samples for biochemical analyses were drawn after at least 10 h of fasting before taking any medication. All biochemical analyses were undertaken using an oxidase-based technique at Roche/Hitachi Modular System (Mannheim, Germany) in the Central Biochemistry Laboratory of the Necmettin Erbakan University Meram School of Medicine.

Quantitative determination of serum immunoglobulins (IgG, IgM, IgA, and IgE) was made through particle-enhanced immunonephelometry using the Siemens BN II/BN ProSpec system (New York, USA). Blood samples obtained from the patients with CVID were taken into 2 mL EDTA tube to evaluate peripheral NK cell subgroups according to CD56 and CD16 expression and CD3, CD4, CD8 expression to determine subtypes T cells. These cells were measured by the BD FACSCanto II 8-color configuration flow cytometer system (California, USA) with fluorescently labeled antibodies. 

The eGFR values of the patients were measured by three different methods. The first method was The modification of diet in renal disease (MDRD) formula: 186 × Serum Cr^–1.154^ × age^–0.203^ × 1.212 (if the patient is black) × 0.742 (if female). In the second technique, eGFR values was measured by Cockcroft–Gault formula: CrCl mL/min = (140 – age) × (weight, kg)** × (0.85 if female)**/(72 × Cr). Lastly, the chronic kidney disease epidemiology collaboration (CKD-EPI) formula was used to calculated eGFR values: 141 × min (Scr/κ (0.7 for females and 0.9 for males))**^α^** ^(–0.329 for females and –0.411 for males)^ × max (Scr/κ)^–1.209^ × 0.993^Age^ × 1.018 (if female) × 1.159 (if black). Urine density, urine ph, fractionated sodium excretion, and serum/urine anion gap were calculated to evaluate the tubule functions of the patients. The normal range for serum anion gap was accepted as > 12 +/– 4 mEq/L, the negative urine gap was accepted as (–20)–(–50) mEq/L [8,9]. Proteinuria in patients with CVID was assessed by 24-h total protein excretion and was defined as an excretion above 150 mg/day in 24-h urinalysis [10].

Complete blood counts with automated differential counts, which included total white blood cells, neutrophils, and lymphocytes, were obtained. The neutrophil to lymphocyte ratio and platelet to lymphocyte ratio were calculated as the ratio of the neutrophils and platelets to lymphocytes, respectively, with both obtained from the same automated blood sample at the onset of the study.

Clinical and experimental data were analyzed using Statistical Package for Social Sciences for Windows v: 15.0 (SPSS Inc., Chicago, Illinois, USA). Descriptive statistics for each variable were determined. Data were expressed as mean ± standard deviation. Results for continuous variables without normal distribution were presented as median (interquartile range (IQR)). A statistically significant difference between the groups was determined by the χ^2^ test for categorical variables. Nonparametric statistics (Mann–Whitney U) and parametric statistics (independent sample t-test) were all used for continuous variables. Associations between the variables were explored using Spearman’s rho test. Linear regression analysis was also performed to define variables associated with proteinuria. A statistically significant difference was considered when p-value ≤ 0.05.

## 3. Results

Demographic, clinical characteristics and biochemical parameters of 27 patients with CVID and 18 healthy control subjects were depicted in Tables 1 and 2. There were no significant differences concerning the following variables between patients and control subjects; sex, age, eGFR values, and serum levels of creatinine. When examined in terms of different methods, eGFR values of both groups, by Cockcroft–Gault, CKD-EPI, and MDRD, were similar. (p: 0.500, p: 0.739, p: 0.753, respectively). Patients with CVID had significantly higher urinary density, fractional excretion of sodium. Also, 24-h proteinuria and metabolic acidosis are found to be higher in patients with CVID when compared to healthy controls (Table 2). When we evaluated the patients with metabolic acidosis for differential diagnosis, we found that there was an acidosis with normal anion gap. In addition, we evaluated patients with acidosis in terms of urinary anion gap results and we found a positive urinary anion gap. 

**Table 1:** Demographic, clinical, and laboratory variables and peripheral lymphocyte subset analysis of patients with CVID.

Demographic and clinical variables of the patients			
Age (year)	39.88 ± 13.47	Sex, F, n (%)	15 (55.6)
Age at diagnosis (year)	32.36 ± 14.86	Diagnostic delay (month)	90 (0–294)
Splenomegaly, n (%)	16 (57.1)	Bronchiectasis, n (%)	15 (53.6)
Laboratory parameters of the patients			
IgG, at diagnosis, (g/L)	2.76 (0.33–6.90)	Neutrophil (103/μL)	3395 (1000–12500)
IgM at diagnosis, (g/L)	0.25 (0.006–5.99)	Lymphocyte (103/μL)	1315 (400–8900)
IgA at diagnosis, (g/L)	0.25 (0.006–1.90)	Platelet (103/mm3)	216607 ± 105563
IgE at diagnosis, (g/L)	17.55 (5–220)	Neutrophil/Lymphocyte ratio	2.84 ± 1.68
CRP (mg/L)	0.73 (0–18.7)	Platelet/Lymphocyte ratio	160.44 ± 121.32
		MPV (fL)	9.80 (6.90–17.40)
Peripheral lymphocyte subset analysis of the patients			
CD3+T cells (%)	78.02 ± 11.74	CD16-56+ NK cells (%)	8.88 ± 6.19
CD4+T cells (%)	35.30 ± 15.07	IgM- CD27+ B cells (%)	1.75 (0–27)
CD8+T cells (%)	36.5 (19–74)		
CD19+B cells (%)	7.35 ± 5.90		

**Table 2:** Comparasion of demografic and laboratory variables of CVID patients and the control group.

Variables	Total	CVID (n: 27)	Control (n: 18)	P values
Age (years)	37.51 ± 11.91	39.88 ± 13.47	33.83 ± 7.97	0.093
Sex, F, n (%)	25 (54.4)	15 (55.6)	10 (55.6)	0.895
GFR (mL/min/1.73m2)	111.45 ± 20.14	109.74 ± 22.56	113.90 ± 15.96	0.500
CKD-EPI	115.58 ± 31.73	123.14 ± 37.23	119.90 ± 21.37	0.739
The Cockcroft–Gault Equation	109.69 (52.6–193.58)	109.89 (52.6–193.58)	108.61 (80.03–144.43)	0.753
MDRD	109.69 (52.6–193.58)	109.89 (52.6–193.58)	108.61 (80.03–144.43)	0.753
Creatinine (mg/dL)	0.78 ± 0.19	0.77 ± 0.21	0.80 ± 0.16	0.573
Urinary pH	5.5 (5.0–7.0)	5.50 (5.0–6.50)	5.5 (5.0–7.0)	0.875
Urinary density (g/mL)	1.017 (1.005– 1.029)	1018.23 ± 5.4	1013.75 ± 5.7	0.014
Metabolic acidosis, n (%)	8 (21.62)	8 (28.6)	0	0.044
24-h urine protein (mg/L)	96.44 (19.04–1635)	136.69 (19.04–1635.4)	75 (25–100)	0.007
FENa(%)	0.76 (0.26–1.86)	0.83 (0.41–1.86)	0.61 (0.26–0.98)	0.014

When patients with CVID were divided into 2 groups according to proteinuria, there were significant differences between the two groups in terms of age and platelet/lymphocyte ratio (p: 0.020 and p: 0.031, respectively) (Table 3).

**Table 3:** Comparision of CVID patients according to the presence of proteinuria.

Variables	Patients with proteinuria(n: 16)	Patients without proteinuria(n: 11)	p
Sex (female), n (%)	7 (43.8)	8 (66.7)	0.229
Age (years)	34.88 ± 13.67	46.54 ± 10.29	0.020
Age at diagnosis (year)	27.75 ± 15.31	38.50 ± 12.27	0.137
Diagnostic delay (month)	101 (0–294)	48 (0–228)	0.100
IgG, at diagnosis, (g/L)	3.60 (1.17–6.90)	1.85 (0.33–6.80)	0.205
IgM at diagnosis, (g/L)	0.27 (0.08–3.16)	0.23 (0.06–5.99)	0.945
IgA at diagnosis, (g/L)	0.29 (0.06–1.90)	0.22 (0.06–1.21)	0.066
IgE at diagnosis, (g/L)	18.35 (5.0–220)	14.20 (5.0–19.0)	0.053
Neutrophil count (103/μL)	3385 (1000–5300)	3510 (1900–12500)	0.347
Lymphocyte count (103/μL)	1145 (400–3800)	1705 (800–8900)	0.074
Lymphopenia (Lymphocyte <1000/mm³), n (%)	6 (37.5)	2 (16.7)	0.227
Platelet count (103/mm3)	211625 ± 78444	193250 ± 137393	0.779
CD3+T cells (%)	77.56 ± 13.02	78.63 ± 10.33	0.816
CD4+T cells (%)	37.0 ± 16.74	33.04 ± 12.86	0.502
CD8+T cells (%)	35 (19–74)	37 (23.70–70)	0.450
CD19+ B cells (%)	5.5 (0–14.0)	7.0 (0–21.0)	0.335
CD16+-56+ NK cells (%)	10.13 ± 6.83	7.21 ± 5.03	0.224
IgM–CD27+B cells (%)	2.65 (0–27)	0.8 (0–11.6)	0.059
Platelet/Lymphocyte ratio	202.56 ± 139.69	104.29 ± 59.15	0.031
Splenomegaly, n (%)	9 (56.3)	7 (43.8)	0.912
Bronchiectasis, n (%)	8 (50)	7 (58.3)	0.662

In the bivariate correlation analysis in patients with CVID, proteinuria was positively correlated with age, CD8+T cells percentage and, negatively correlated with albumin and platelet/lymphocyte ratio (Table 4).

**Table 4:** Bivariate correlation results between proteinuria and other parameters in patients with CVID.

Parameters	rS	P value
Age (years)	0.496	< 0.001
CD8+T cells (%)	0.427	0.02
Platelet/Lymphocyte ratio	–0.585	0.01
Albumin (g/dL)	–0.642	< 0.001

We also performed a linear regression analysis to define variables that are independently associated with proteinuria (Table 5). Age, albumin, CRP, CD8+T cells, and platelet/lymphocyte ratio were included in this model. Albumin, CRP, and CD8+ T cell percentage were found to be the independent predictor of proteinuria.

**Table 5:** Variables of proteinuria in patients with CVID.

Parameters	Standardized beta coefficients	t	P value	95% CI
Step 1				
Age (years)	0.015	0.110	0.913	–6.001–6.671
CD8+T cells (%)	0.275	2.168	0.042	0.272–13.152
Albumin (g/dL)	–0.373	–2.341	0.029	–572.686–33.836
CRP (mg/L)	0.455	2.990	0.007	3.225–17.959
Step 2				
CD8+T cells (%)	0.275	2.228	0.036	0.464–12.998
Albumin (g/dL)	–0.380	–2.682	0.014	–548.252–70.055
CRP (mg/L)	0.453	3.084	0.005	3.449–17.605

## 4. Discussion

There were 3 main findings of the present study. First, renal dysfunction including proteinuria, metabolic acidosis, and urinary excretion of sodium and urine density was higher in patients with CVID. Second, age and CD8+T cells percentage are positively correlated with proteinuria. Lastly, low albumin, high CRP, high CD8+ T cell percentage are found to be independent predictors of proteinuria in patients with CVID.

In 1993, Hermaszewski et al. [11] presented the study that first mentioned renal involvement and complications in patients with immune deficiency. In this study in which 240 patients with CVID were evaluated, only 5 patients had renal involvement. Of these, 2 patients had chronic kidney disease, 1 patient had nephrotic syndrome and 2 patients had the nephritic syndrome. Later, in different studies, many conditions such as renal granuloma, focal segmental glomerulonephritis, membranous nephropathy (MN), membranoproliferative glomerulonephritis (MPGN), renal amyloidosis, nephrotic syndrome, various tubulopathies, and end-stage renal failure were also identified in patients with CVID [12–18]. In 2014, Sarkar et al. [19] found findings compatible with chronic tubulointerstitial nephritis, such as tubular degeneration, interstitial fibrosis, and atrophied tubules due to lymphomononuclear cell infiltration, when the patient with CVID evaluated by biopsy due to renal failure. Similarly, Capistrano et al. [18] demonstrated the presence of impaired tubular function in patients with CVID, such as decreased urinary concentration and decreased acidification capacity. In our study, we found that the number of patients with proteinuria and metabolic acidosis was higher in patients with CVID than the control group. In addition to these findings, FENa was significantly higher than the control group, and in patients with metabolic acidosis, the serum anion gap was in the normal range, while the urine anion gap was not negative. These results show that patients with humoral immune deficiency are also at risk for interstitial and tubular renal diseases. This situation can be explained by increased inflammation and T cell infiltration [18]. Also, patients with metabolic acidosis have a urinary ph value of 5.5 or below, suggesting a proximal tubulopathy in these patients. In this context, close follow-up of patients especially for diseases such as osteomalacia, rickets, and osteoporosis due to impaired proximal tubule functions might be required. 

There are many reasons such as infection, immune dysfunction, autoimmunity, delay in diagnosis and treatment, capillary leakage syndrome, and environmental toxins that can increase chronic ongoing inflammation in CVID patients [20]. In healthy individuals, albumin is a late reacting negative acute-phase protein and is reabsorbed by the proximal tubule [21]. In patients with proteinuria, protein leakage activates a series of the detrimental intracellular signaling cascade in tubular cells. As a result, an inflammatory microenvironment is created by the hyperproduction of numerous chemocytokines to increase the migration of immune cells [22]. It has been claimed that permeability factors, which are produced due to primary T cell disorder in patients with CVID and cause disruption in glomerular podocyte function, may contribute to the development of nephrotic syndrome [23,24]. Also, when CD8+ T cells are activated, almost all cells express MHC class I molecules, so they have the potential to cause tissue damage. Therewithal activated CD8+ T cells can produce very high levels of tumor necrosis factor (TNF) and IFN-γ that can contribute directly and/or indirectly to target cell destruction in autoimmune diseases [25]. In the present study, we found a positive correlation between proteinuria and CD8+T cells. In addition to this data, we found CRP and CD8+T cells as predictors of proteinuria in linear regression analysis. These findings may suggest that proteinuria occurs as a result of increased inflammation. 

Platelet/lymphocyte ratio is an easy-to-calculate, repeatable, and simple measurement with predictive properties in acute inflammation and thrombotic events [26]. Increased platelet/lymphocyte ratio is associated with disease activity in some malignancy, rheumatological disease, and psychiatric disorder [27–30]. However, unlike these data, in our study, patients with proteinuria had a statistically lower platelet/lymphocyte ratio. This difference may be due to the frequent occurrence of autoimmune cytopenias due to immune dysregulation in patients with CVID. As is known, immune dysregulation in patients with CVID usually manifests with immune thrombocytopenia [31–33]. Also, thrombocytopenia may be due to splenic sequestration or autoimmunity in addition to immune dysregulation. Our group reported that low platelet count is a risk factor for bronchiectasis in patients with CVID [34]. In our study, platelet values were found to be lower in the group with proteinuria, although not statistically significant. Therefore, the negative correlation between platelet/lymphocyte ratio and proteinuria may be caused by thrombocytopenia. This result may be an indirect indication that immune dysregulation or autoimmunity causes impaired kidney function.

It is obvious that as the disease duration and patient age increases, patients will be exposed to inflammation for a longer period. Although there is a marked decrease in the frequency of infection with immunoglobulin replacement therapies (IGRT) [35], the risk of developing gastrointestinal infections and/or complications, malignancy, and granulomatosis disease is not affected by IGRT [36,37]. Besides, with increasing age; renal load increases due to the risk factors such as contrast imaging, the use of immunosuppressive drugs (such as steroid, cyclosporine, and nonsteroidal antiinflammatory drugs). Therefore, these patients become more sensitive to kidney disorders. In our study, patients with proteinuria were significantly older than patients without proteinuria. And, in statistical analysis, we found a positive correlation between age and proteinuria in patients with CVID.

In conclusion, in the present study, we demonstrated that patients with CVID have a higher risk of nephropathy and proteinuria. Our findings suggest that nephropathy may be more associated with tubulopathy, while we observed that proteinuria is associated with increased inflammation. Therefore, we think that patients who are followed-up with a CVID diagnosis should be monitored for nephropathy and related complications. It should also be considered that proteinuria may be an indirect indicator of increased inflammation in these patients. Further randomized and controlled studies evaluating the kidney functions in patients with CVID are needed.

## Informed consent

The study protocol was approved by the Ethics committee of the Necmettin Erbakan University (Date: 16.11.2018; approval number: 2018/1574). Informed consent was obtained from study participants.
